# Specialized Pro-Resolving Lipid Mediators: Endogenous Roles and Pharmacological Activities in Infections

**DOI:** 10.3390/molecules28135032

**Published:** 2023-06-27

**Authors:** Fernanda S. Rasquel-Oliveira, Matheus Deroco Veloso da Silva, Geovana Martelossi-Cebinelli, Victor Fattori, Rubia Casagrande, Waldiceu A. Verri

**Affiliations:** 1 Laboratory of Pain, Inflammation, Neuropathy, and Cancer, Department of Pathology, Center of Biological Sciences, Londrina State University, Londrina 86057-970, Paraná, Brazil; fernandarasquel@gmail.com (F.S.R.-O.); matheus.deroco@gmail.com (M.D.V.d.S.); geovana.martelossi@uel.br (G.M.-C.); 2 Vascular Biology Program, Boston Children’s Hospital, Department of Surgery, Harvard Medical School, Boston, MA 02115, USA; 3 Department of Pharmaceutical Sciences, Center of Health Science, Londrina State University, Londrina 86038-440, Paraná, Brazil

**Keywords:** SPMs, inflammation, resolution, bacteria, virus, parasites

## Abstract

During an infection, inflammation mobilizes immune cells to eliminate the pathogen and protect the host. However, inflammation can be detrimental when exacerbated and/or chronic. The resolution phase of the inflammatory process is actively orchestrated by the specialized pro-resolving lipid mediators (SPMs), generated from omega-3 and -6 polyunsaturated fatty acids (PUFAs) that bind to different G-protein coupled receptors to exert their activity. As immunoresolvents, SPMs regulate the influx of leukocytes to the inflammatory site, reduce cytokine and chemokine levels, promote bacterial clearance, inhibit the export of viral transcripts, enhance efferocytosis, stimulate tissue healing, and lower antibiotic requirements. Metabolomic studies have evaluated SPM levels in patients and animals during infection, and temporal regulation of SPMs seems to be essential to properly coordinate a response against the microorganism. In this review, we summarize the current knowledge on SPM biosynthesis and classifications, endogenous production profiles and their effects in animal models of bacterial, viral and parasitic infections.

## 1. Introduction

Physiological response of the body to an infection consists in orchestrating a complex immunological defense, including triggering the inflammatory process. Acute inflammation is characterized by the production and release of molecules such as cytokines, chemokines, metalloproteinases, prostaglandins, and leukotrienes, which attract leukocytes, mainly neutrophils and macrophages, at the inflammation site [1,2]. Inflammatory mediators are essential to fight the pathogen, but may be detrimental to the host tissue, especially when inflammation becomes chronic. The “resolution” phase of the inflammation happens to prevent collateral damage and it is an active process: the so-called Specialized Pro-resolving lipid Mediators (SPMs) are key players produced by the metabolism of polyunsaturated fatty acids (PUFAs): arachidonic acid (AA), eicosapentaenoic acid (EPA), docosapentaenoic acid (DPA) and docosahexaenoic acid (DHA). SPMs are currently classified into lipoxins (LX), resolvins (Rv), maresins (MaR), and protectins (PD) [3].

SPMs act as immunoresolvents by sending “stop signals” within the picogram to nanogram dose range [4] in a time- and context-dependent manner [5]. These compounds have also been described to act in a tissue- and disease-specific manner [6]. As a result of that, SPMs control the influx of granulocytes to the site of the inflammation, stimulate microbe killing and phagocytosis of cell debris and pathogens, limit pain, activate tissue-resident cells that promote repair [6,7,8,9], and lower antibiotic requirement [10].

Infectious diseases account for one in four deaths worldwide and represent one of the major causes of organ/tissue impairment due to both pathogen and uncontrolled inflammation-induced tissue damage [11,12]. In this sense, significant effort is constantly being made to find antimicrobial therapies that modulate the inflammatory process, avoid antibiotic resistance, stimulate innate and adaptive immune responses, and have low or no toxicity, reducing mortality.

Given that chronic inflammation is implicated in several diseases, there is a growing interest in discovering new pro-resolving mediators and elucidating how they act to promote resolution. In this review, we summarize and discuss the current knowledge on the biosynthetic pathways and classification regarding SPMs, including the ways in which production of these mediators occurs upon an infection, pointing out the main cell types, signaling molecules and pathways involved in this process. We also compile pre-clinical and clinical studies that have investigated the effect and/or levels of SPMs in infectious diseases.

## 2. SPM Biosynthesis

**Figure 1:**
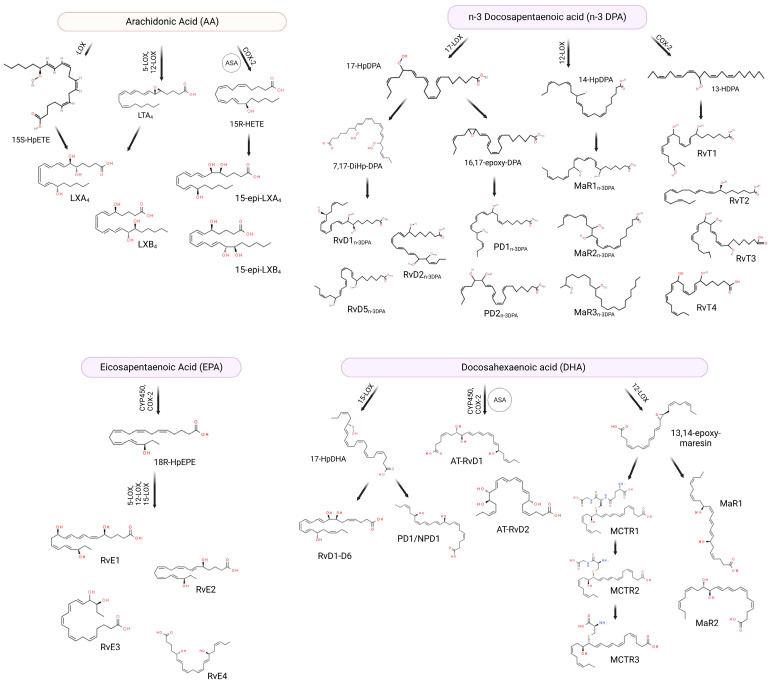
SPMs can be biosynthesized from omega-3 and omega-6 fatty acids through different enzymes. Arachidonic acid (AA) originates lipoxins (LXs) and 15-epi lipoxins (15-epi-LXs), eicosapentaenoic acid (EPA) originates E-series resolvins (RvE), omega-3 docosapentaenoic acid (n-3 DPA) can be converted into D-series resolvins_n-3 DPA_, protectins _n-3 DPA_, maresins _n-3 DPA_, and 13-series resolvins (RvTs), while docosahexaenoic acid (DHA) can be transformed into D-series resolvins (RvDs), protectins (PDs), aspirin-triggered D-series resolvins (AT-RvDs), maresins (MaRs) or maresin conjugate tissue regeneration (MCTRs). ASA: acetylsalicylic acid. Created using BioRender.com (accessed on 17^th^ April 2023).

Resolution is an active and highly regulated process [13]. A class switch from pro-inflammatory mediators such as leukotrienes (LT) and prostaglandins (PG) to SPMs drives resolution of the inflammatory response. To promote their immunoresolvent actions, SPMs bind to specific G protein-coupled receptors (GPCR) expressed by several cells [14,15], which are summarized in Table 1. SPMs are synthesized endogenously from the metabolization of omega-3 (i.e., DHA, EPA, and DPA) PUFAs or omega-6 PUFAs (i.e., AA) (Figure 1) [16]. DHA can be converted into D-series resolvins (RvD; RvD1, RvD2, RvD3, RvD4, RvD5, and RvD6) [17,18,19], protectins (PD1)/neuroprotectins (NPD1) [20,21], and maresins (MaRs; MaR1 and MaR2) [22]. EPA, on the other hand, is converted into the E-series resolvins (RvE; RvE1 and RvE2) [23]. There is an additional series of SPMs formed by the presence of aspirin, which have been coined aspirin-triggered SPMs [24]. Figure 1 presents a schematic representation of the SPM synthetic pathways and chemical structures.

RvDs are formed from conversion of DHA through two lipoxygenations. First, DHA is converted to (17*S*,4Z,7Z,10Z,13Z,15E,19Z)-17-hydroperoxydocodahexaenoic acid (17*S*-HpDHA) through the action of 15-LOX in the carbon-17 (C-17) position. It then undergoes a second lipoxygenation by the same enzyme in C-7, generating an intermediate peroxide which can be reduced forming RvD5 or transformed into 7*S*, 8*S*-epoxide. 7*S*, 8*S*-epoxide can further undergo enzymatic hydrolysis generating RvD1 and RvD2. Alternatively, the second lipoxygenation may occur in the C-4 position forming another peroxide intermediate, which is similarly converted to RvD3, RvD4 and RvD6 [15,17]. There are also aspirin-triggered (AT) RvDs, such as AT-RvD1 and AT-RvD2—the difference in biosynthesis is in the initial lipoxygenation of C-17, which occurs in the presence of aspirin through acetylated COX-2 or via cytochrome P450 [25]. RvEs, in turn, are generated from the oxygenation of the EPA by acetylated COX-2 or via cytochrome P450. This oxygenation generates the intermediate acid 18*R*-hydroperoxy-eicosapentaenoic (18*R*-HpEPE), which is transformed into 18*R*-hydroxyEPA (18*R*-HEPE) by the action of a peroxidase [23]. Subsequently, 5-LOX promotes the lipoxygenation of 18*R*-HEPE into hydroperoxide, which can be transformed into epoxide and hydrolyzed into RvE1 [15] or may be reduced by means of a peroxidase in RvE2 [26]. Alternatively, the 18*R*-HEPE intermediate can undergo lipoxygenation through the action of 12-LOX or 15-LOX, becoming converted into 17,18-diHEPE, also called RvE3 [27]. Furthermore, EPA can be converted into 15*S*-HpEPE through lipoxygenation by 15-LOX, subsequently reduced to 15*S*-HEPE by peroxidase. The 15*S*-HEPE intermediate undergoes a second lipoxygenation by 5-LOX, becoming converted into 15*S*-hydroxy-5*S*-HpEPE (15*S*-H,5*S*-HpEPE), which is reduced to RvE4 through the action of a peroxidase [28,29].

Also synthesized from DHA, PD1 and NPD1 (when produced in neural tissues) are formed from the lipoxygenation of 17*S*-HpDHA through 15-LOX [20,30], generating the epoxide intermediate 16(17)-epoxydocosatriene. This intermediate is subsequently converted into PD1/NPD1 through the action of a hydrolase [31]. Additionally, maresins are synthesized from lipoxygenation that occurs in C-14 through 12-LOX, forming the 14*S*-hydroperoxiDHA (14*S*-HpDHA), which undergoes a second lipoxygenation by the same enzyme and forms the intermediate epoxide 13*S*,14*S*-epoxy-maresin [31]. This intermediate is subsequently converted into MaR1 and MaR2 by means of the action of a hydrolase or a soluble hydrolase, respectively [31]. Additionally, 13*S*,14*S*-epoxy-maresin can be converted into maresin conjugates in tissue regeneration (MCTRs). MCTR1 (13*R*-glutathionyl, 14*S*-hydroxy-4*Z*,7*Z*,9*E*,11*E*,13*R*,14*S*,16*Z*,19*Z*-docosahexaenoic acid) is catalyzed by both leukotriene C4 synthase (LTC4S) and glutathione S-transferase Mu 4 (GSTM4). γ-glutamyl transferase (GGT) converts MCTR1 in MCTR2 (13*R*-cysteinylglycinyl, 14*S*-hydroxy-4*Z*,7*Z*,9*E*,11*E*,13*R*,14*S*,16*Z*,19*Z*-docosahexaenoic acid) that, in turn, can be transformed into MCTR3 (13*R*-cysteinyl, 14*S*-hydroxy-4*Z*,7*Z*,9*E*,11*E*,13*R*,14*S*,16*Z*,19*Z*-docosahexaenoic acid) by a dipeptidase enzyme [32].

In addition to DHA and EPA, arachidonic acid, derived from the enzymatic oxygenation of omega-6, is also involved in the biosynthesis of SPMs, more specifically in the synthesis of the lipoxin family (LX; LXA_4_ and LXB_4_) and aspirin-triggered lipoxins (ATLs) [33]. As previously mentioned, a lipid mediator class switch is necessary to begin resolution, and that happens by polymorphonuclear cells (PMNs) reducing production of LT and PG and that of increasing SPMs such as LX [14]. For LX synthesis to occur, cell–cell interaction is necessary. This occurs through a process known as transcellular biosynthesis, and can be achieved through two main pathways [34]. The first pathway occurs through lipoxygenation of AA in C-15 by 15-LOX, forming 15*S*-HpETE, which is secreted from cells (i.e., eosinophils, monocytes, and epithelial cells). 15*S*-HpETE is then converted into 5,6-epoxytetraene by the action of 5-LOX in PMNs and monocytes and subsequently hydrolyzed into LXA_4_ and LXB_4_ [34]. The second pathway occurs in an LTA*_4_*-dependent manner. Initially, the conversion of AA to LTA*_4_* occurs through the action of 5-LOX, which is secreted and subsequently absorbed by adherent platelets. In platelets, LTA*_4_* is transformed into LXA_4_ and LXB_4_ by 12-LOX [34,35]. In addition to the two main pathways, alternative pathway may occur where the presence of aspirin acts on COX-2, redirecting its catalytic activity and promoting the formation of LX. The initial acetylation of COX-2 by aspirin promotes the formation of 15*R*-hydroxyeicatetraenoic acid (15*R*-HETE), which is converted into 15-epimeric-LXs (15-epi-LXs) by the action of 5-LOX called aspirin-triggered lipoxins [24,34,36].

There are also reports in the literature of SPMs like RvD, PD and MaR being biosynthesized from docosapentaenoic acid (n-3 DPA), an intermediate metabolite in the conversion of EPA to DHA [53]. Among these SPMs are PD1_n-3 DPA_, PD2_n-3 DPA_, MaR1_n-3 DPA_, MaR2_n-3 DPA_, MaR3_n-3 DPA_, RvD1_n-3 DPA_, RvD2_n-3 DPA_, and RvD5_n-3 DPA_ [53,54]. At the site of the inflammatory response, n-3 DPA is converted into the intermediate 17-hydroperoxy-8*Z*,10*Z*,13*Z*,15*E*,19*Z*-docosapentaenoic acid (17-HpDPA) through the action of 17-LOX, acting as a substrate for the formation of SPMs [53]. First, this intermediate can be transformed into the intermediate epoxide 7,17-dihydro(peroxy)-DPA, which is sequentially converted into RvD5_n-3 DPA_ or into the 7,8-epoxy,17-hydroxy-DPA molecule; both actions are performed via 5-LOX. Finally, 7,8-epoxy,17-hydroxy-DPA can be converted into RvD1_n-3 DPA_ and RvD2_n-3 DPA_, also by lipoxygenation by 5-LOX [53]. Furthermore, 17-HpDPA can be transformed into the epoxide intermediate 16-17-epoxy-DPA, which is enzymatically hydrolyzed into PD1_n-3 DPA_ and PD2_n-3 DPA_ [53]. Alternatively, n-3 DPA can be converted to the intermediate 14-hydroperoxy-7*Z*,10*Z*,12*E*,16*Z*,19*Z*-docosapentaenoic acid (14-HpDPA) through the action of 12-LOX. This intermediate can undergo a second lipoxygenation, becoming converted to MaR3_n-3 DPA_, or it can be converted to the intermediate epoxide 13,14-epoxy-DPA, which is enzymatically hydrolyzed to MaR1_n-3 DPA_ and MaR2_n-3 DPA_ [53]. In addition, n-3 DPA can generate 13-hydroxy-docosahexaenoic acid (13-HDPA) through COX-2, and neutrophils are able to convert 13-HDPA via lipoxygenation to the 13-series resolvins RvT1, RvT2, RvT3 and RvT4 [55]. To date, studies addressing n-3 DPA-derived SPMs are not as numerous as those for EPA- or DHA-derived SPMs. The lack of data makes it difficult to compare the potency of n-3 DPA-derived SPMs with that of EPA- or DHA-derived SPMs. The effects of n-3 DPA-derived SPMs, however, seem to be generally comparable to those of other SPMs [53,56].

Among the lipoxygenases involved in the biosynthesis of SPMs mentioned above, 15-LOX (ALOX15), mainly the 15-LOX-1 isoform, plays an important role in the pathways by catalyzing the initial transformation reactions of PUFAs [57]. LOX are found in organisms from two of the three domains, namely Bacteria and Eukarya [58]. In this context, it is important to understand the existence of orthologous enzymes between species (i.e., mice, rats, and humans). In humans, there are six LOX genes (ALOX5, ALOX15, ALOX15B, ALOX12, ALOX12B and ALOXE3) that encode six different isoforms of LOX. In comparison, mice have orthologs for all human ALOX isoforms [59]. However, these orthologs vary in their specificity, exhibiting differences in enzymatic oxygenation. For example, human ALOX15 acts enzymatically as 15-LOX, whereas murine ALOX15 acts enzymatically as 12-LOX [57,60,61]. Furthermore, human ALOX15 is expressed in epithelial cells, red blood cells and eosinophils, in addition to monocytes/macrophages and neutrophils through induction by interleukins (IL). In mice, on the other hand, it is found in resident macrophages [57,62]. Studies have shown that this difference in lipoxygenation from 12-LOX to 15-LOX improves the ability to synthesize LX, optimizing the process of inflammation resolution [61]. However, when it comes to DHA oxygenation, both ALOX15 (human and murine) produce similar amounts of 17-HDHA and 14-HDHA [60], showing similar actions.

SPMs actively induce the inflammation resolution process [63] by reestablishing tissue homeostasis, increasing host defense, and interfering with the maintenance of pain signals [15]. In addition, they promote the cessation of the influx of PMNs [23], reduce the production of pro-inflammatory mediators [24,64] and induce phagocytic activity of macrophages [65]. Failures in anti-inflammatory actions may contribute to the development of chronic inflammation [65,66]. Some factors, such as age, sex, and race, can influence the formation of SPMs and their pro-resolution abilities. In humans, aging contributes to the development of a heightened inflammatory state and decline in physiological functions, characterized by increased tumor necrosis factor (TNF)-α, nuclear factor kappa B (NF-κB), IL-1β and IL-6 [67]. In this context, studies demonstrate that elderly mice have reduced local levels of PMNs and increased levels of PMNs in inflammatory exudates, delaying the resolution process [65]. Regarding sex and race, one study with 53 participants found that after myocardial infarction (MI), plasma levels of metabolites from AA and DHA were higher in white individuals of both sexes (female and male) than those in Black male and female individuals. EPA levels were higher in white males than in white females and Black individuals of both sexes [68]. Regarding endogenous SPM levels, RvE1 was significantly lower in Black patients, while PD1 levels were lower in white, male patients [68]. Despite disparities in lifestyle (such as physical exercise and diet) that are suggested as the cause of higher incidence of MI among Black individuals [69], distinct SPM signatures may provide a better understanding and clinical guidance on personalized therapies in the future.

### Cross-Linking Pro-Inflammatory and Pro-Resolving Mediator’s Biosynthesis

It is well described that some mediators of inflammation with contrasting activities share common precursors: one example is LX and PG that share arachidonic acid as the substrate during enzymatic conversions. It is also true that metabolization of one substrate can be performed by multiple enzymes, and the same enzyme can convert different substrates into multiple metabolites, sometimes with divergent actions. So, what regulates this process during inflammation?

While this question cannot be fully answered with the current available experimental data, some evidence suggests that there are substrate preferences for each enzyme. Conversion of omega-3 and omega-6 happens by oxidation by lipoxygenases, cyclooxygenases, or the cytochrome P450 oxidase/epoxygenases. Arachidonic acid is the preferred substrate for COX-2 [70,71], originating pro-inflammatory mediators. COX-2 oxygenates EPA at about 45% the rate of AA [71], despite AA and EPA displaying Km values similar to those of COX-2, individually [72]. When aspirin (ASA) acetylates COX-2, however, it prevents the formation of prostanoids, favoring the lipoxygenase-type reaction, generating 15*R*-HETE from AA, that can be metabolized into 15-epi-LXs [73].

Lipoxygenases, one of the main types of enzymes in SPM biosynthesis, accept AA, DHA and EPA as substrates, but with differences: 15-LOXs preferentially converts DHA > EPA > AA, while 12*S*-LOX’s preference is DHA > EPA > AA and that of 5-LOX’s is AA and 5*S*-HpETE [74]. In fact, human lipoxygenases have different kinetics with each metabolite serving as a substrate, which are deeply discussed by Kahnt et al. [75]. 

The third route of metabolization of PUFAs is through cytochrome P450, or CYP450. The first double bond on C3, present in omega-3 but not omega-6, is a preferred site of epoxidation catalyzed by CYP450 [76]. Most CYP isoforms have EPA as preferred substrate, while AA and DHA are converted at similar rates [76]. Examples of isoforms with higher rates of conversion of EPA over AA and DHA are CYP2J2 and CYP2C23 isoforms present in human heart and rat kidney, respectively [77,78]. 

Another factor that dictates SPM production over pro-inflammatory biosynthesis is that, depending on the activation status of macrophages on the inflammatory foci, these cells change the expression of lipoxygenases: macrophages that differentiate to the M2-like phenotype by IL-4 or IL-13 or, upon efferocytosis of apoptotic cells, upregulate the expression of 15-LOX-1 [79,80,81,82]. In addition, monocytes and macrophages minimally express 15-LOX-2 unless these cells undergo long-term stimulation by zymosan or lipopolysaccharide (LPS) via toll-like receptor activation [80,83]. In parallel with M2 polarization, PGE*_2_* production decreases [84,85].

Additionally, SPM receptor expression likely influences regulation of resolution versus inflammation: a study published by Krishnamoorthy and colleagues [86] showed that less RvD1 is required to lower neutrophil migration in human ALX/FPR2-overexpressing transgenic mice. Proper or stimulated expression of receptors for SPMs in order to maximize beneficial effects during resolution seems to be decisive, but further evidence is needed. 

As the same enzymes operate for both omega-3 and omega-6 conversion pathways, the bioavailability ratio of EPA, DHA and AA during inflammatory process may be critical, which further supports the intake of EPA- and DHA-rich diets to increase SPM production. There are several phospholipases A2 (PLA2s) responsible for remodeling the cell membrane that directly impacts PUFA bioavailability and metabolism. Evidence suggests that certain phospholipases also have preferences: human cytosolic cPLA2 prefers AA, calcium-independent iPLA2 prefers EPA, and secreted sPLA2 selectively prefers DHA as a substrate [87]. Secreted PLA2 group IID (PLA2G2D) appears to be linked to pro-resolution activity, and it is preferentially expressed by macrophages and dendritic cells [88]. Another study observed that DHA enrichment of mononuclear cell membranes was directly correlated with phospholipases D (PLD) activation by DHA [89]. 

It is important to highlight that each experimental condition is unique, and these results may not be fully extrapolated to the context of infections since not all results described above were observed upon pathogen stimuli. However, these data bring potential approaches to be investigated in order to potentiate SPM production and effectively induce resolution.

## 3. SPM Levels in Patients with Infectious Diseases

There are a limitingly small number of clinical studies on SPM levels in patients with infectious diseases. However, this also highlights the need of further studies in this field. Despite not being fully elucidated, a growing body of evidence correlates better clinical prognosis and better survival rates with increased levels of SPMs. Therefore, in this section, we discuss the current data on how SPMs levels can affect the evolution of the clinical picture of patients with infections.

### 3.1. Bacterial Infections

In a study led by Jesmond Dalli [90], the authors quantified the serum levels of LTB_4_, PGE_2_α and pro-resolving mediators RvD1, RvD2, and PD1 in patients with sepsis. It was found that non-survivors had lower levels of pro-resolution mediators, especially in the chronic phase of the disease, than survivors. It has also been demonstrated that the profile of lipid mediators can be directly related to the severity of the sepsis condition; patients with marked levels of pro-inflammatory lipid mediators have a marked and exaggerated response to the microorganism, resulting in worse prognoses. In addition, surviving patients needed smaller amounts of antibiotics since they had a more efficient immune response. The authors also point out that the severity of sepsis is directly associated with platelet aggregation, as this factor is directly linked to organ failure and death [90]. It is believed that high levels of AT-RvD1, AT-RvD3, and AT-PD1 may decrease the expression of COX-2 and LTB_4_. Hence, proper pro-resolving mediator production seems to regulate a balanced immune response and correlate with better survival rates, as the cytokine storm present during sepsis can be as detrimental as the infection.

In another study, plasma from 66 patients with sepsis in intensive care units and 20 healthy subjects (controls) were analyzed [91]. Sepsis patients were grouped into survivors or non-survivors, depending on their outcome on day 28 of the study. Pro-inflammatory and pro-resolutive mediators were detected: cytokines IL-6 and IL-8 were higher in concentration on non-survivors when compared to controls, while LXA_4_ and Annexin A1—pro-resolution mediators that inhibit leukocyte migration and eicosanoid production—levels were lower in survivors and non-survivors compared to the control patients [91]. In individuals with tuberculosis, AA-derived pro-inflammatory lipids were abundantly present, such as PGE_2_, LTB_4_ and PGF_2_α. Among the most prominent SPMs detected, there were LXB_4_ and 5*S*,15*S*-diHETE [92]. Indeed, infections trigger the generation of prominent inflammatory mediators, but the host capacity to correctly produce endogenous pro-resolving molecules that actively regulate the response also has a significant role in establishing or a chronic inflammatory process, or resolving it. In this sense, further investigation is needed to explore the correlation between higher circulating SPM levels and better outcomes.

As DHA and EPA, precursors of some SPMs, are present in plants and animal sources, consumption of omega-3 rich foods impact bioavailability of these precursors to form SPMs [93]. To investigate how omega-3 supplementation could affect the levels of PUFAs and oxylipins—metabolites of PUFAs that can be converted into SPMs—upon endotoxin (LPS) challenge in men, Walker and colleagues [94] designed a randomized crossover study. Blood samples were collected at baseline, 1, 2, 4 and 8 h after the challenge. The authors observed that EPA and DHA levels were increased in the omega-3-treated group by 432% and 142%, respectively, when compared to controls. Some oxylipins, such as HEPEs (from EPA), were strongly increased [94]. In a similar study, adults challenged with endotoxin and treated with omega-3 presented higher levels of circulating 18-HEPE, 17-HDHA, AT-LXA_4_, LXB_4_, RvE1 and RvD1 [95]. Despite the small number of participants in both studies, the results provide initial evidence of lipid profile modulation by the presence of LPS and how omega-3 bioavailability alters the synthesis of pro-resolution mediators during bacterial infections.

### 3.2. Viral Infections

The COVID-19 pandemic highlighted the importance and urgency of understanding the impact of this infectious disease on the immune system. To compare serum levels of pro-inflammatory molecules and SPMs between SARS-CoV-2 patients and healthy subjects, Turnbull and colleagues performed a lipidomic analysis where 44 bioactive lipids were quantified [96]. The SARS-CoV-2 infection was associated with a significant mobilization of both pro-resolutive and pro-inflammatory mediators’ production when compared to matching controls, as increased levels of LTB_4_, PGE_2_, 5-HETE, 13-HODE and 17-HDHA were detected [96]. In bronchoalveolar lavages, this strong sign of immune response was also observed—levels of LTB_4_, PGE_2_, DHA, n-3 DPA, RvD1, RvD2, RvD4, RvD5, PDX, 17-HDPA_n-3_, 14-HDHA and 17-HDHA were markedly boosted in COVID-19 subjects when compared to the non-infected group [97].

This increase, though, may differ in patients that are more affected by this infection. In a cohort study conducted in Beaumont Hospital, Ireland, 38 patients who tested positive for the SARS-CoV-2 virus had their plasma lipid mediators profile analyzed. Comparing two main groups, critically ill (patients that required invasive mechanical ventilation) and severe disease cohorts (the ones that required supplemental oxygen or non-invasive ventilation), a downregulation of the 5-lipoxygenase (*ALOX5*) pathway in critically ill patients who had lower levels of RvD1 and RvD3 was reported. Additionally, severe disease patients had higher concentrations of PD1_n-3 DPA_ and MCTR1, and both cumulative SPM concentration and ratio of SPM concentration to pro-inflammatory mediators indicate that increased SPM production is linked to better outcomes [98]. Comparably, serum from severe patients showed a significant increase in RvE1 and MaR2 [99]. Moreover, COVID-19 alters the activation and function of circulating phagocytes, as PD1, RvT1, RvE3, and 10*S*,17*S*-diHDPA positively correlate with phagocytic ability of monocytes and neutrophils [100].

The possible mechanisms by which SPMs would improve COVID-19 outcomes are speculated elsewhere [101,102,103,104]. Recent evidence shows that the SARS-CoV-2 virion spike 1 glycoprotein (S1) can induce cytokine and chemokine release by macrophages and modulate microRNAs miR-103, miR-16 and miR-29a, known to control the inflammatory responses. Interestingly, in vitro treatment with RvD1 and RvD2 promoted resolution by lowering S1-induced production of IL-8, TNF-α, MCP-1, augmented macrophage phagocytic activity and regulated miRNAs expression to reduce IKK/NF-κB activation and downstream signaling cytokines [105].

This variation in SPM production among differently affected patients may not be restricted to COVID-19. Lipidomic profiling of nasal washes from patients with influenza, categorized in low, medium and high clinical scores, revealed that samples from individuals with a high clinical score and elevated levels of cytokines/chemokines also presented notable higher percentages of PGE_2_, LTE_4_, and mediators from the lipoxygenase, DHA, and EPA pathways [106].

Altogether, these data indicate that elucidating how each SPM is produced during different disease profiles may help clinicians to better understand a patient’s prognosis and plan effective therapies in the future. Moreover, using supervised machine-learning methodologies, a study highlighted that increased plasma levels of RvD4, 10*S*,17*S*-diHDPA, 15*R*-LXA_4_, and MaR1 are linked to therapy responsiveness in rheumatoid arthritis patients [107]. This might suggest a potential biomarker role for SPMs in the context of disease-modifying anti-rheumatic drug (DMARD) responsiveness [107]. These results might provide important disease monitoring information to clinicians, but also could be extrapolated to other diseases as well, including infectious ones.

## 4. SPM Regulation of Infection in Animal Models

**Figure 2:**
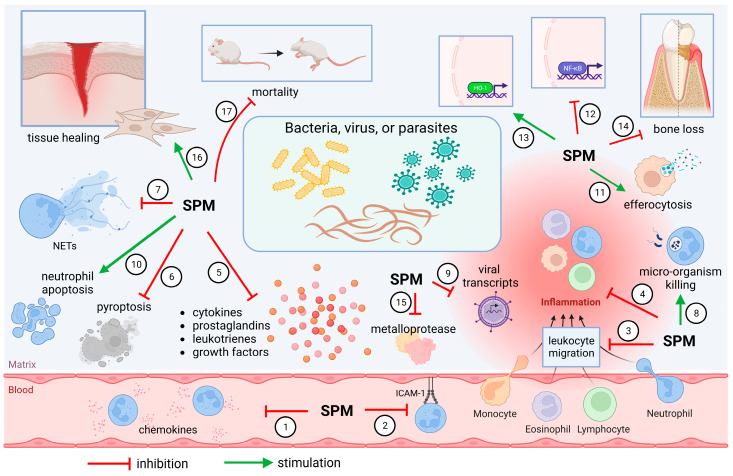
SPMs control infection by different mechanisms. SPMs limit chemokine release (1) [120,125,133,136] and ICAM-1 expression (2) [115], reducing migration of leukocytes (3) [110,113,114,117,118,120,123,125,127,129,132,134,136,142] to the inflammatory site. Systemic inflammation (4) is also diminished [109,119], together with prominent reduction in pro-inflammatory molecules’ release (5) [10,120,121,124,125,128,130,133,134,136,137,139,141]. SPMs also decrease pyroptosis (6) [118] and NET formation (7) [132], without compromising microbicidal activities (8 and 9) [10,112,113,114,120,124,126,127,129,131,132,133,135,141]. Neutrophil accumulation is avoided also by stimulating neutrophil apoptosis (10) [110,123] and clearance of cell debris by macrophages (11) [123,126,130]. Inhibition of NF-κB (12) [129,134,137,138] and upregulation of HO-1 (13) [115] favor inflammation control. As a result, less bone loss is observed (14) [117,119,122], and tissue architecture is preserved by reducing the activity of metalloproteases (15) [121,125,136] and stimulating tissue healing (16) [119,122,128]. Improvement of survival was also observed (17) [109,110,112,116,118,120,124,129,131,133,135,137,141]. Created using BioRender.com (accessed on 6 April 2023).

During an infection, it is essential that the host immune system regulates the production and release of pro-inflammatory and pro-resolutive mediators to maintain the integrity of tissues and cells while it fights pathogens. This proper balance leads to homeostasis instead of chronic inflammation. Thus, SPMs have an important role regulating inflammatory infection in many animal models (Figure 2), as previously demonstrated. In general, SPMs limit the neutrophil-mediated tissue [4] in addition to increasing the repair capacity of macrophages [108]. Therefore, for a better understanding of their mechanisms, we divide the actions of SPMs into bacterial, viral, and parasitic animal models of infections, as summarized on Table 2 and Figure 2. 

### 4.1. Bacterial Infections

SPMs are known to control bacterial infections, and bacteria or their products can shape the production of SPMs in different steps of the inflammatory response. 

Interestingly, if not by bactericidal effect per se, SPMs stimulate the phagocytosis and clearance of different pathogens. For instance, RvD1 synergizes with ciprofloxacin to promote the non-phlogistic phagocytosis of *Pseudomonas aeruginosa* during lung infection [140] and against *Escherichia coli* [10]. Similarly, other SPMs such as RvD2 [124], MaR1 [141], or PDX [133] decrease local and systemic bacterial burden, which leads to increased survival in a model of sepsis [82]. In this section, we review the literature on the possible outcomes of SPMs applied as treatments in disease models caused by bacteria.

#### Effects of SPMs in Animal Models of Bacterial Infections

One of the most extensively studied models of infectious disease is sepsis, a severe worldwide health concern and a leading cause of disability and mortality. Improper host immunological response to pathogens and excessive inflammation can lead to multi-organ disfunction and death [143]. Therefore, adequate innate and adaptive immune defense against the microorganism allied with a controlled inflammatory process is the key to reduce severity of sepsis. Due to SPMs ability to induce both features, bioactive lipid mediators have been studied in models of induced sepsis, mainly using cecal ligation and puncture (CLP), that results in a systemic polymicrobial infection that mimics sepsis in humans. LXA_4_ treatment significantly reduced mortality of CLP rats. Despite not affecting phagocytic activity, bacterial load was reduced. This was accompanied by reduced IL-6, monocyte chemotactic protein 1 (MCP-1) and IL-10 levels, in addition to inhibition of NF-κB activation in peritoneal macrophages [112]. Likewise, in a different study, LXA_4_ controlled neutrophil migration while increasing the phagocytic ability of the ones that were able to migrate to the inflammatory foci [113,114]. The authors observed that apoptotic, bacterial clearance and phagocytic activities were induced without uncontrolled free radical production [113]. Moreover, LXA_4_ can affect the virulence of *Pseudomonas aeruginosa* by acting as an antagonist and partial agonist of LasR, an important transcription factor that coordinates production and release of pathogenic factors of *P. aeruginosa* [114]. Treatment with LXB_4_ reduced inflammation and improved survival of mice after CLP by limiting neutrophil infiltration and protecting cells from pyroptosis [118]. In a model of *E. coli*-induced sepsis, 15-epi-LXA_4_ presented a synergic effect with antibiotics by regulating IL-6 and TNF-α production by macrophages, thus limiting bacterial replication, neutrophil migration, and resulting in better survival rates [109]. Administration of LXA_4_ during the late phase of *Klebsiella pneumoniae*-induced pneumosepsis reduced mortality significantly by ablating excessive inflammation and bacterial load [116]. 

Resolvins also demonstrated promising results in treating sepsis. Following CLP, mice that received RvD1 treatment had lower numbers of bacteria in blood and peritoneal fluid, and also inhibited uncontrolled neutrophil migration and NF-κB activation. RvD1 also diminished the rate of apoptosis of CD3^+^ T cells of the thymus [129], which is a relevant cause of immunosuppression that is highly detrimental during sepsis. Another member of the resolvins family, RvD2, exerted pro-resolutive effects upon CLP by lowering local and systemic bacterial burden, reducing neutrophil and increasing mononuclear peritoneal counts. This increase in macrophages was also correlated with improvement of phagocytosis of *E. coli* by macrophages as well as changes on IL-17, IL-10, PGE_2_, IL-6, IL-1β, IL-23, TNF-α, PGE_2_, and LTB_4_ levels [124].

Xia et al. [133] demonstrated that PDX also had an impact on sepsis outcomes after CLP. Following treatment with PDX, the authors observed improvement in survival rates, prevention of multiple-organ injury (as demonstrated by liver and kidney function markers), reduced bacterial colony formation unit (CFU) counts from both blood and peritoneal fluid, and suppressed neutrophil recruitment while increasing macrophage numbers with higher phagocytic abilities. Cytokine production and polarization of macrophages were also affected by PDX, as M2 macrophages (F4/80^+^CD206^+^) were increased and IL-6, TNF-α and MCP-1, markers of M1 macrophages, were decreased after treatment [133]. Similar promising results were attributed to MaR1 that effectively reduced lactate, acetate, and pyruvate levels in serum of CLP mice, downregulating proinflammatory cytokines and NF-κB, mitigating mitochondrial damage of lung tissues and resulting in better survival ratios [137,138]. 

Airway infections are caused by bacteria, fungus, or viruses, which can be spread through direct or indirect contact and eventually increase the risk of a secondary coinfection [144,145,146]. Respiratory tract infections gained particular attention in the last few years after the SARS-CoV-2 global pandemic. Commonly, prominent inflammation is one of the hallmarks of airborne infections, which leads to significant tissue injury. Hence, pre-clinical models are being extensively employed to study the role of SPMs on pathogen-induced airway diseases. Using a sepsis-induced acute lung injury (ALI) model, the authors determined that PDX ameliorated histopathological changes in lung tissue, reduced bacterial load, pulmonary edema, PMN migration, and production of IL-1β, IL-6, TNF-α, and MCP-1. Mechanistically, these effects were likely linked to suppression of NF-κB and upregulation of PPARγ, a natural receptor of PUFAs with regulatory role in the inflammatory process [134]. In mice, hydrochloric acid aspiration plus administration of *E. coli* mimics aspiration pneumonia in patients, one of the leading causes of ALI and acute respiratory distress. Treatment with 100 ng RvE1 promoted bacterial clearance, lowered PMN counts and several proinflammatory markers on lung homogenates. Inhibition of NF-κB and survival improvement were also observed [120]. 

In a slightly distinct model of pneumonia, induced by *E. coli* and *P. aeruginosa*, AT-RvD1 reduced CFU counts alone and potentialized ciprofloxacin activity when combined with it. Moreover, neutrophil numbers on bronchoalveolar fluid were reduced by AT-RvD1 but not by ciprofloxacin alone. Promotion of efferocytosis by macrophages also contributed to a better disease outcome [126]. Similar results were found by Isopi et al. [127]—RvD1 promoted resolution of *P. aeruginosa* infection and inflammation in cystic fibrosis. In addition, the Gram-negative bacterium *Haemophilus influenzae* is an opportunistic pathogen that can infect the upper respiratory tract and exacerbate inflammation in susceptible patients. AT-RvD1 alters the inflammatory cell profile and promotes efferocytosis of apoptotic neutrophils, dampening COX-2, IL-6, and TNF-α in a murine nontypeable *H. influenzae* infection model [130].

Pre-clinical models of peritonitis have been employed to study antimicrobial and/or anti-inflammatory therapies for several decades. It is an important and reproducible approach to investigate infections [147,148]. Using an *E. coli*-induced peritonitis model, the authors observed a protective effect of RvD2 by promoting PMN apoptosis, limiting neutrophil migration and enhancing macrophage phagocytic ability, effects that were absent in mice deficient in GPR18, an RvD2 receptor [123]. Similarly, RvD1 shortened the resolution interval of *E. coli* peritonitis, and when combined with antibiotic therapy, significantly raised bacterial phagocytosis and reduced IL-1β and IL-6 [10]. The culture of human neutrophils with RvE1 also enhances phagocytosis of opsonized *E. coli* [149]. When RvD1, RvD5 and PD1 (50 ng) were combined with a suboptimal dose of ciprofloxacin, bacterial titers were diminished and mice were protected from hypothermia. Additionally, levels of IL-6 and granulocyte-macrophage colony-stimulating factor (GM-CSF) were reduced, and the production of the precursor of D-series resolvins, 17-HDHA, was increased [10]. These results highlight a promising ability to potentiate antibiotic efficacy. A combination of these three pro-resolving mediators as a treatment at endogenous levels induced phagocytosis of *E. coli* by human macrophages in vitro, and pro-inflammatory genes related to the expression of NF-κB and TNF-α were downregulated by RvD5 through activation of a GPR32 receptor [10]. In a different study, after *E. coli* intraperitoneal injection, treatment with LXA_4_ was able to induce neutrophil apoptosis through phosphorylation of the BCL-2-associated death promoter (BAD) and reduced expression of the myeloid leukemia sequence 1 (MCL1) anti-apoptotic protein [110]. Additionally, treatment with RvE1 can decrease MPO activity and production of IL-1β, and IL-6, and increase bacterial clearance [120]. 

Intestinal infection by the enteropathogenic *Escherichia coli* (EPEC) is a leading cause of mortality among infants. The Gram-negative bacteria *Citrobacter rodentium* shares many pathogenic mechanisms with EPEC in humans, and is therefore used as a model of intestinal infection in newborn rodents. Therapeutic effects of RvD1 and RvD5 were observed in *C. rodentium* infection, showing substantial decrease in bacterial load, lessening neutrophil influx and rescuing 33–100% of infected infants from death (depending on the number of CFU injected). In addition, the treated groups of neonates developed serum IgG responses comparable to those of infected adults, suggesting a remarkable impact of RvD1 and RvD5 on immunological memory establishment [131].

Periodontitis is an inflammatory disease primarily caused by a microbiota dysbiosis and raising of a pathobiont with excessive proliferation and invasion of oral cavity, destroying periodontal tissues; it is commonly associated with the onset of systemic illnesses if left untreated [150,151,152]. Experimental periodontitis shares pathogenic features with other inflammatory diseases such as septic arthritis and is employed to study inflammation and bone loss in oral cavity, which are easily observable. In rabbits, topical application of an LXA_4_ analog prevented loss of connective tissue and alveolar bone and significantly diminished inflammatory infiltrate [117]. Restoration of soft tissues and resolution of the intense inflammation were attributed to RvE1, in contrast to PGE_2_ and LTB_4_ administration which worsened the disease. In addition, RvE1 reduced bone resorption and serum IL-1β and C-reactive protein (CRP), which are markers of systemic inflammation [119]. Similar results were observed on a periapical periodontitis model in rats treated with RvD2, as well as healing of periapical lesions and lower bacterial burden [122]. 

Despite *Staphylococcus aureus* being a commensal microorganism of the body, it can become opportunistic and cause skin and articular infections. Commonly, murine skin pouch models are employed to assess *S. aureus*-induced infections. Coadministration of RvD2 and *S. aureus* via intra-pouch injection reduced bacterial titers and neutrophil numbers [123]. The combination of PD1, RvD5 and RvD1 reduced bacterial load by 10-fold; strikingly, a combination of PD1, RvD5 and RvD1 with suboptimal doses of vancomycin reduced bacterial load by 100-fold. This demonstrates that, in parallel with classic antibiotic strategies, SPMs may be suitable as a promising approach against pathogens.

Neutrophil extracellular traps (NETs) are scaffolds of chromatin released by neutrophils along with proteases and enzymes that help entrap invading microorganisms [153]. Excessive NET production is linked to collateral tissue damage [154]. During murine *S. aureus* infection, RvT1, RvT2, RvT3 and RvT4 reduced bacterial titers and NET formation, and in vitro, the treatment stimulated NET clearance by human monocyte-derived macrophages, promoting resolution [132]. This points to another mechanism by which SPMs could control tissue destruction caused by inflammatory products.

In addition, growing evidence highlights the importance of time-regulated production of SPMs. An interesting study published by Sordi and colleagues [116] demonstrated that LXA_4_ played a detrimental role when administered at an early phase of sepsis, worsening the infection, versus a protective effect when administered during the late phase of the disease. Additionally, distinct outcomes were observed when administering RvD2 at different timepoints in an *E. coli* peritonitis model. A higher number of apoptotic PMN were detected when treatment was provided during the peak of the inflammation, but no such effect was observed when RvD2 was administered during the onset of inflammation [123]. Therefore, these studies guide our attention to certain particularities of each context, timepoint of administration and disease profile that should be considered (and deeply explored) in future research to maximize SPMs’ beneficial potential. 

Altogether, these effects stimulated by SPMs could be helpful because they might contribute to the reduction in new bacterial antibiotic resistance mechanisms given the pro-efferocytosis actions of SPMs. Moreover, some antibiotics, such as β-lactams, induce bacteriolysis releasing LPS and lipoteichoic acid (LTA) that can induce post-infection sequelae due to persistent activation of immune cells [155]. Therefore, the ability of SPMs to enhance bacterial clearance, lower antibiotic requirements, and shorten resolution time interval [10] can be essential to uncover new ways to treat infection. Ultimately, these effects can contribute to reduce antibiotic resistance and post-infectious sequelae.

### 4.2. Viral Infections

The span and magnitude of the immune response against viruses depends on how the virus interacts with host cells, the stages of replication, dissemination, and infection. Humoral immunity assumes that viruses or infected cells stimulate B lymphocytes to produce antibodies aimed at neutralizing and/or opsonizing the infected cell. The binding between antibodies with the virus/infected cell can block the interaction of the virus with the host cell, as well as facilitate recognition by cells of the immune system, especially by cytotoxic cells, and activate the lysis of the infected cell by the complement system [156]. Cell-mediated immunity, on the other hand, assumes that the infected cells will be recognized by cells of the immune system, through the recognition of MHC molecules, molecules related to DAMPs cell damage, or through the production of cytokines by the infected cells. Among the main cells involved in this mechanism are cytotoxic and helper T lymphocytes as well as NK cells [156]. Many viruses capable of causing chronic infections tend to activate dendritic cells and macrophages, stimulating them to produce TGFβ and IL-10, and these cytokines limit the inflammatory response against the virus [156,157,158,159,160].

#### Effects of SPMs in Animal Models of Viral Infections

Many studies involving viral infections and SPMs investigate the interaction of mediators with the influenza virus [102]. The current understanding is that there is a direct relationship between the virulence of the virus strain and the production of SPMs [102] since strains with high virulence (H5N1 and H1N1) can reduce lipoxin levels. It was previously demonstrated that this decrease caused by H5N1 occurs mainly due to the inhibition of ALOX5, the gene of the enzyme responsible for the synthesis of lipoxins, and that its inhibition results in the dissemination of the virus to other tissues, since the reparative tissue response was compromised [161]. PD1 was also previously demonstrated to be effective against influenza, improving mice survival rates, pulmonary functions, and infection by inhibiting nuclear export of viral transcripts [135]. In addition, SPMs may help humoral immunity against viruses. The production of IgG was stimulated by LXB*_4_* in B cells collected from individuals vaccinated against influenza, and the authors concluded that the mechanism involved was related to the increase in COX2 induced by LXB*_4_*, which in turn led to an increase in BLIMP1 and XBP1 expression in B cells. Both are plasmatic cell differentiation factors that are directly linked to the activity of memory B cells [162].

Respiratory syncytial virus (RSV) is the most common cause of viral pneumonia among children. In mice, lung inflammation caused by RSV was mitigated by MaR1, with significant increase in viral clearance and amphiregulin production, an epithelial growth factor that aids in the resolution process [139]. These effects were, at least in part, attributed to MaR1 ability to modulate aberrant inflammatory regulatory T cells (Tregs) that express FoxP3 and signal through LGR6, as LGR6-deficient mice presented high viral load and exacerbated type-2 immune response [139]. Both macrophages and Tregs express LGR6 constitutively [139].

In a murine stromal keratitis model of ocular infection by herpes simplex virus (HSV)-1, untreated eye lesions do not regress even after viral clearance due to excessive local inflammatory process and Th1/Th17 cells influx. Corneal ulceration, edema and neovascularization arise from the local inflammatory response. Combined with excessive infiltration of neutrophils, macrophages, dendritic and natural killer cells [163], it may result in blindness [164]. Rajasagi and collaborators demonstrated that, in mice, the administration of RvE1 [121] or AT-RvD1 [125] minimized the disease severity by limiting leukocyte migration, neovascularization and production of pro-inflammatory cytokines, contributing to lesion healing. Also, the AT-RvD1 treatment upregulated IL-10 production, a major regulator of T-helper cell activation and secreted by resolution type macrophages in response to RvE1 [165,166] and suppressing STAT1, which influences in Th1 cell differentiation and IFN-γ expression [121,125]. Comparable results were described after topical application of NPD1, which mitigated the severity of the disease, dampening leukocyte infiltration, as well as cytokine and chemokine (IL-6, CXCL1, CXCL-10, CCL-20), metalloproteinases (MMP-2 and MMP-9) and vascular growth factor (VEGF-A) production, therefore controlling tissue destruction and abnormal vascularization [136]. Thus, SPMs may represent a safe and interesting addition to current therapies to mitigate viral infections.

### 4.3. Parasitic Infections

Diseases caused by protozoan parasites and helminths still account for an enormous social and health impact in tropical regions of the world, costing billions of dollars annually [167]. The pathogenesis of parasitic infections is complex and differ by pathogen. For example, eggs and larvae frequently induce granuloma formation and fibrosis, protozoans trigger Th1 response with high levels of IFN-γ and TNF-α, and helminths induce a strong Th2 response with significant eosinophilia [168]. Furthermore, parasitic infections often feature acute or chronic neuroinflammation and are linked to an assortment of clinical outcomes, as pro-inflammatory cytokines released by microglial cells and astrocytes are key players of the pathological process [169]. The presence of parasites can impair the activity of glial cells, and this is commonly related with pro-inflammatory mediators’ levels, cytotoxic action of nitric oxide, and reactive oxygen species [169]. When the infection becomes chronic, persistence of the pathogen leads to tissue damage and perpetuates inflammatory processes, markedly characterized by cellular infiltrate, composed mainly of T cytotoxic and T helper lymphocytes, macrophages, and B cells [170].

#### Effects of SPMs in Animal Models of Parasitic Infections

Although limited, some studies have addressed the role of SPMs as treatments for parasitic infections. Cerebral malaria, caused by the parasite *Plasmodium berghei* in mice, is mitigated by 15-epi-LXA_4_ [111]. The pathogenesis observed in this animal model resembles infection by *P. falciparum* in humans. The relevance of lipid mediators produced by 5-LOX, including LXA_4_, was demonstrated by the infection of mice deficient in this enzyme (Alox5-deficient mouse) that presented intense lymphocyte infiltration and high pro-inflammatory cytokine expression, and also accelerated mortality. These parameters were ameliorated by the LXA_4_ epimer, 15-epi-LXA_4_ [111]. In another study, Souza and colleagues [115] described protective effects of LXA_4_ on cerebral malaria by reducing vascular dysfunction and edema, inducing hemeoxigenase-1 (HO-1) expression and improving survival percentage. A distinct—but not mild—parasitic disease caused by *Trypanosoma cruzi*, Chagas disease, can lead to heart failure and death, if neglected [171]. A serious clinical manifestation of this disease is chronic cardiomyopathy, with significant focal leukocyte infiltration and fibrosis. Mice infected with *T. cruzi* received AT-RvD1, antiparasitic therapy or a combination of both; AT-RvD1 alone reduced serum levels of IFNγ and IL-1β, along with inflammatory infiltrate and molecular markers of cardiac hypertrophy and tissue fibrosis. Moreover, AT-RvD1 alone or in combination with an antiparasitic drug reduced parasitic load [128]. This indicates that, similarly to what happens in bacterial infections, SPMs could be used in combination to current clinically active drugs to improve their efficacy.

### 4.4. What Mechanisms Are Shared by SPMs upon Infection Caused by Different Pathogens?

When comparing the described effects of SPMs between different types of pathogens (e.g, bacteria vs. virus), some similarities can be noticed. As described above, these pro-resolution mediators are able to limit the production and release of chemokines [120,125,133,136], resulting in fewer cells recruited to the inflammatory site [110,113,114,117,118,120,123,125,127,129,132,134,136,142] and strongly downregulate pro-inflammatory cytokine release [10,120,121,124,125,128,130,133,134,136,137,139,141], without compromising the microbicidal capacity of phagocytes [10,112,113,114,120,124,126,127,129,131,132,133,135,141] that is an essential feature during an infection. As a consequence of controlled inflammation, tissue healing is improved [119,122,128] and survival rates are higher [109,110,112,116,118,120,124,129,131,133,135,137,141]. 

On the other hand, differences in SPM effects between different types of pathogens are harder to point out. This is due to two main reasons: first, the immunopathology mechanisms of infections can differ tremendously from each other, and naturally, different aspects are considered and measured in each study. Therefore, there are some knowledge gaps due to a lack of comparable data. And second, each SPM has different chemical structures and kinetics, and their activity can be similar or different depending on the context. Hopefully, novel studies will help fill this gap in the future.

## 5. Endogenous Production of SPMs in Animal Models of Infection

In addition to the studies evaluating the effect of exogenous (administrated) SPMs in different disease contexts, endogenous lipid mediator levels have been quantified upon infection in certain animal studies, as summarized in Table 3. 

### 5.1. Production of SPMs in Animals upon Bacterial Infection

While SPMs have been shown to control *E. coli* infection, as discussed above, *E. coli* is also known to induce the production of SPMs by different cells. In a self-resolving model of the *E. coli* infection, endogenous RvD3 [172] and MaR1 [173] were detected in inflammatory peritoneal exudates (24 h and 4 h after infection, respectively), indicating that production of these mediators is induced upon infection. Similarly, after stimulus with *E. coli*, mouse and human vagus nerves produce SPMs to contribute to host homeostasis [174]. The human vagus nerve produces RvD4, RvD6, MaR1, 4*S*,14*S*-diHDHA, and 15-epi-LXA_4_ while the mouse vagus nerve produces only PDX [174], showing a species-specific production of SPM upon *E. coli* infection. This is in accordance with a study showing that vagotomy delays resolution of self-limiting *E. coli* infection by controlling the production of PD conjugate in tissue regeneration (PCTR1) and PD1 [175]. Therefore, in self-resolving *E. coli* infection, bacteria stimulate the vagus nerve to produce SPM that have a role in resolution of inflammation and infection. The role of the vagus nerve is biologically relevant since vagotomy delays resolution by impairing SPM production.

Acetylcholine derived from neurons acts on CD335^+^ILC3 to increase the production of the PCTR1 pathway marker 17-hydroxy-4*Z*,7*Z*,10*Z*,13*Z*,15*E*,19*Z*-docosahexaenoic acid (17-HDHA). Additionally, adoptive transfer of ILC3 in *E. coli*-infected and vagotomized mice restore resolution index by reducing neutrophil recruitment and shortening resolution interval [175], demonstrating the relevance of vagus nerve–ILC3 communication. Expanding this cross-talk, in case of depletion of ILC3 or in vagotomized mice, peritoneal macrophages produce lower levels of PCTR1, indicating a role for ILC3–macrophage cross-talk in PCTR1 production. Reduction in peritoneal ILC3, furthermore, results in impaired *E. coli* phagocytosis, increased peritoneal bacterial load, and increased inflammation-initiating eicosanoids PGD_2_, PGE_2_, PGF_2_α, TXB_2_ and LTB_4_ [175]. In accordance, adoptive transfer of ILC3 treated with a lipoxygenase inhibitor abrogates the beneficial effect of these cells, indicating that ILC3 needs active lipoxygenase likely to produce SPMs, which then actively induce peritonitis resolution and host responses to infections [175]. Altogether, these data not only show the immunoresolvent properties of SPMs but also that cellular communications exist through the release of SPM. These cellular communications can be both from immune cell to immune cell and from neuron to immune cell. These types of cellular communications use SPMs to regulate infection outcome.

Pneumonia caused by *E. coli* in mice resulted in temporal synthesis and release of AT-RvD1, detected 24 h and peaking 72 h after infection, as determined by metabololipidomics performed on lung tissue [126]. In a similar model of pneumococcal pneumonia in mice, blockade of ALX/FPR2 (lipoxins and some D-series resolvins receptor) increased pulmonary edema, bacterial burden, and protein accumulation on air spaces [179]. This indicates that impairment of the resolution phase of the inflammation may contribute to disease worsening and progression to chronic disease. In fact, upregulation of the production of SPMs may be a protective mechanism exerted by beneficial microorganisms—the probiotic bacteria *Clostridium butyricum* MIYAIRI 588 helps protect the gut epithelial barrier from damage caused by antibiotic treatment by upregulating PD1, palmitoleic acid and 15d-prostaglandin J_2_ [176]. Furthermore, in animals lacking a nucleotide-binding domain, leucine-rich repeat-containing receptor, pyrin domain-containing-3 (NLRP3) inflammasome, CLP-induced sepsis resulted in decreased mortality due to reduced proinflammatory mediators and augmented LXB_4_ generation [118]; in addition, *M. tuberculosis*-infected mice produce LXA_4_ to enhance control of the infection [180].

Although not in a mouse model of infection induced by bacteria, α-Hemolysin (Hla) from *S. aureus* induces SPM formation. Intraperitoneal injection of Hla increases production of 15-lipoxygenase-1 (15-LOX-1) enzyme in murine M2-like macrophages. This effect is correlated to an increase in Hla phagocytosis and depletion of Hla impaired SPM formation, indicating that recognition of Hla by macrophages is important to promote bactericidal effects [181]. Interestingly, it was also noted that a particular threshold of bacterial challenge is required to activate different mediator pathways: 15-LOX-1-mediated SPM production required higher *S. aureus* multiplicity of infection (MOI) than that for 5-LOX or COX products. Also, SPM levels were detected later than COX/5-LOX products (starting at 90 min and 30 min, respectively) [181]. This suggests that different pathways may be activated during the response against *S. aureus* to promote distinct lipid mediator production.

### 5.2. Production of SPMs in Animals upon Viral Infection

Unfortunately, there is limited evidence on the quantification of SPM levels in animal models of viral infections. Animals that undergo severe influenza infection show increased levels of COX pathway products, such as PGE_2_, and decreased levels of DHA metabolites: 17-HDoHE—resolvin and protectin precursor—PD1, and LXA_4_ in lung tissue. Interestingly, these metabolites showed a large impact on the viral replication in vitro and on survival rates, when administered, in mice [135]. Similarly, 17-HDoHE was identified in murine samples following administration of sublethal doses of different influenza viruses, PR8/H1N1 (high pathogenicity) and X31/H3N2 (low pathogenicity). In the same study, different forms of hydroxylated DHA were detected in specific stages of the infection: 4-, 10-, 13-, and 20-HDoHE levels increased between days 3 and 5 after infection with lethal doses of PR8/H1N1, while 8-, 14-, and 16-HDoHE peaked at day 3 [106]. The importance of temporal identification of SPMs and their precursors relies on the fact that contrasting results have been reported on pro-resolving mediator administration in early versus late phase of the infection, highlighting the relevance of completely elucidating the way in which resolution is orchestrated as well as the beneficial SPM treatment administration time frame [116,123].

### 5.3. Production of SPMs in Animals upon Parasitic Infection

As previously mentioned, since SPMs are endogenous players of inflammation resolution, regulation of production of these mediators in a timely manner is essential. Parasitic infections are not different. Cytokines such as TNFα, IFN-γ and IL-12 are essential for host resistance against the protozoan *Toxoplasma gondii*. During the early phase (5–10 days after inoculation), IL-10 seems to be the main player regulating IL-12-dependent IFN-γ production, but after IL-10 levels decrease, there is a substantial production of LXA_4_, beginning at day 10 and reaching a plateau at day 15 after infection, limiting excessive cytokine production and mortality during a late phase of the infection. Therefore, LXA_4_ seems to work in synergy with IL-10, as the mouse strain deficient in the enzyme that produces LXA_4_, 5-LOX^−/−^ succumb two weeks after inoculation [177]. In another article, the authors inoculated *Toxocara canis* and *Toxocara cati* eggs in mice to induce neurotoxocarosis, a common and underestimated parasitic infection that affects populations globally [178]. Temporal analysis of bioactive lipid metabolites during the infection showed a significant increase in LOX-derived metabolites when compared to noninfected group. In addition, significantly elevated levels of NPD1 were detected at days 14, 28 and 42 after infection (p.i.) in *T. canis*-infected and at days 14 and 42 p.i. in *T. cati*-infected mouse brain. In the cerebellum, increased levels of NPD1 were additionally detected on day 98 (*T. canis*) and day 28 (*T. cati*) [178]. Neuronal cell survival, control of leukocyte infiltration, oxidative stress protection, and inhibition of COX-2 expression and NF-κB activation are some of the effects of NPD1 in the brain [182,183]. These studies contribute to understanding the temporal production profile of these mediators and the ways in which they coordinate resolution, infection control and healing.

In this manner, these data suggest that impairment of the resolution phase of the inflammation, either by compromising cell signaling, pathogen recognition by the host or disruption of temporally regulated SPM production and release are directly related to worsening disease progression and outcomes.

## 6. Conclusions

Management of diseases caused by pathogens have been a challenge for centuries, and infections are one of the leading causes of morbidity and mortality globally. Factors that contribute to this problem can be pathogen-related, such as antibiotic resistance and virulence factors, or host-related, such as a lack of basic resources to prevent or treat infections and a debilitated immune response (due to comorbidities, stress, habits, etc.), and all directly impact morbidity and mortality of infections. In fact, perpetuation of the inflammatory response is commonly associated with high-prevalence neurodegenerative, cardiovascular, and rheumatic diseases [184]. A previous assumption was that resolution of inflammation would be a passive process; however, it is currently understood as an active process which is induced by SPMs.

As key players of resolution, SPMs are produced during self-resolving sterile and non-sterile inflammation. During infections, accumulating evidence shows that endogenous production of pro-resolving mediators correlates with lower cytokine levels and is linked to a better prognosis in patients [90,98], and disruption of SPM production or signaling negatively impacts microbicidal response in animals [175,179]. Pre-clinical studies point out the bacterial clearance capacity of SPMs [10,112,113,114,120,124,126,127,129,131,132,133,141], reduction in leukocyte influx [110,113,114,117,118,120,123,125,127,129,132,134,136,142] and enhanced phagocytosis/efferocytosis [113,114,118,123,124,126,130,133]. These activities are tissue- and disease-specific [6], and temporal regulation of SPM production seems to be essential to orchestrate a beneficial instead of a deleterious response [116,123]. We have summarized the SPM mechanisms in Figure 2.

Along with proper elucidation of the ways in which SPMs act during infections, future research is needed to address current limitations in this field. First, new families of SPMs are being discovered, and the receptors they may act on still need to be investigated. Second, orthologs from different species need to be explored and part of lipid mediator profiling in pre-clinical studies may not be transferable to humans [185]. There are a few interventional clinical trials involving SPMs or their precursors (ClinicalTrials.gov Identifier: NCT02719665; NCT01675570; NCT04088240; NCT04308889; NCT01865448); unfortunately, none of them involve infectious diseases and all lack results and conclusions thus far. This fact limits the discussion of the clinical pharmacological perspectives of SPMs in infections. Third, development of analogs with increased biostability may overcome challenges and help uncover physiologic roles of these mediators, so SPMs can become useful tools as biomarkers and effective treatments soon. Fourth, lipidomic studies are extremely useful; however, it seems that we also need information about the profile of SPM receptors and the cells that are expressing those receptors. Evidence has determined that enhancing SPM receptor increases the final biological activity without alteration of SPM concentration [186]. This indicates that in addition to the levels of SPMs, the profile of receptor expression is essential information to understand their contribution to disease outcomes. Therefore, knocking out specific receptors in selected cell types would be an essential approach to bring definitive evidence on the endogenous roles of SPMs in infections and other diseases. In any case, the pharmacological use of SPMs is also a valid approach evidencing the therapeutic application of exogenous SPMs administration.

## Figures and Tables

**Table 1:** Receptors for SPMs and their cellular expression.

Precursor	SPM	Receptor		Expressed by	Reference
		Human	Mice		
AA	LXA_4_	ALX/FPR2	ALX/FPR2	Neutrophils, eosinophils, macrophages, monocytes, NK cells, innate lymphoid cells (ILCs)	[37,38]
	Aspirin-triggered LXs (ATLs)	ALX/FPR2	ALX/FPR2	Neutrophils, eosinophils, macrophages, monocytes, NK cells, innate lymphoid cells (ILCs)	[37,38,39,40]
EPA	RvE1	BLT1, ERV1/ChemR23	BLT1, ERV1/ChemR23	Neutrophils, macrophages, eosinophils, monocytes, dendritic cells, lymphocytes, mast cells, NK cells, innate lymphoid cells (ILCs)	[38,41,42,43]
	RvE2	BLT1	BLT1	Neutrophils, macrophages, eosinophils, monocytes, dendritic cells, lymphocytes, mast cells	[38,42,43]
n-3 DPA	RvD5_n-3 DPA_	GPR101	GPR101	Macrophages, neutrophils, monocytes	[44]
DHA	RvD1	ALX/FPR2, DRV1/GPR32	ALX/FPR2	Neutrophils, eosinophils, macrophages, monocytes, lymphocytes, NK cells, innate lymphoid cells (ILCs)	[37,38,45,46,47]
	RvD2	DRV2/GPR18	DRV2/GPR18	Macrophages, monocytes, neutrophils	[38,48]
	RvD3	ALX/FPR2, DRV1/GPR32	ALX/FPR2	Neutrophils, eosinophils, macrophages, monocytes, lymphocytes, NK cells, innate lymphoid cells (ILCs)	[38,40,49]
	RvD5	DRV1/GPR32	Unknown	Macrophages, neutrophils, monocytes, lymphocytes	[10,38]
	PD1/NPD1	GPR37	GPR37	Macrophages	[50]
	MaR1	LGR6	LGR6	Macrophages, neutrophils, monocytes	[51,52]

LXA4: Lipoxin A4; FPR2: N-formyl peptide receptor 2; ALX: LX A4 receptor; DRV1/GPR32: G-protein-coupled receptor 32; RvE1: Resolvin E1; BLT1: Leukotriene B4 receptor 1; ERV1/ChemR23: Chemerin chemokine-like receptor 1; RvE2: Resolvin E2; RvD1: Resolvin D1; RvD2: Resolvin D2; RvD3: Resolvin D3; DRV2/GPR18: G-protein-coupled receptor 18; PD1: Protectin D1; NPD1: Neuroprotectin D1; GPR37: G-protein-coupled receptor 37; MaR1: Maresin 1; LGR6: Leucine-rich repeat-containing G-protein-coupled receptor 6.

**Table 2:** Pharmacological activities of SPMs on animal models of infection.

SPM	Dose	Animal Model		Infectious Agent	Effect/Outcome	Ref.
15-epi-LXA_4_	1 μg/animal	C57BL/6 mice	Sepsis	*E. coli*	↓ systemic inflammation ↑ survival	[109]
	200 μg/kg	Balb/c mice	Acute lung injury	*E. coli*	↓ number of neutrophils ↓ edema ↑ survival	[110]
	1 µg/animal (LXA_4_ and 15-epi-LXA_4_)	C57Bl/6J mice	Cerebral malaria	*P. berghei*	↓ mortality ↓ accumulation of CD8 + IFN-γ+ cells	[111]
LXA_4_	40 μg/kg	Sprague–Dawley rats	Sepsis (CLP)	Polymicrobial	↓ bacterial load ↑ survival	[112]
	7 μg/kg	Sprague–Dawley rats	Sepsis (CLP)	Polymicrobial	↓ neutrophil migration ↑ neutrophil phagocytic ability ↓ bacterial load	[113,114]
	0.5 μg/kg	C57Bl/6J mice	Cerebral malaria	*P. berghei*	↑ HO-1 expression ↓ capillary congestion and endothelial disfunction ↓ ICAM-1 expression	[115]
LXA_4_ analogs	100 μg/kg (LXA_4_ analog), 2.5 μg/kg (LXA_4_)	Swiss mice	Pneumosepsis	*K. pneumoniae*	↑ improvement of the survival rate when administered on later phase of sepsis	[116]
	5–6 μg/2–3 μL/tooth	Rabbits	Periodontitis	*P. gingivalis*	↓ leukocyte infiltration ↓ bone loss	[117]
LXB_4_	1 μg/animal	C57Bl/6J, Nlrp3^−/−^, Asc^−/−^, P2rx7^−/−^, Casp7^−/−^, and Il18^−/−^ mice	Sepsis (CLP)	Polymicrobial	↑ survival ↓ leukocyte migration ↓ pyroptosis	[118]
RvE1	4 μg/tooth	Rabbits	Periodontitis	*P. gingivalis*	↑ healing of tissue and bone ↓ systemic inflammation ↓ C-reactive protein levels	[119]
	100 ng/animal	C57Bl/6J mice	Pneumonia and acute lung injury	*E. coli*	↓ neutrophil accumulation ↑ bacterial clearance ↓ IL-1β, IL-6, HMGB-1, MIP-1α, MIP-1β, MCP-1 ↓ mortality	[120]
	300 ng/eye	Balb/c mice	Stromal keratitis	Herpes simplex virus-1 (HSV-1)	↓ angiogenesis ↓ lesions ↓ IL-6, IFN-γ, IL-17, KC, VEGF-A, MMP-2 and MMP-9 ↑ IL-10	[121]
RvD2	20ng	Wistar rats	Periapical periodontitis	Polymicrobial	↑ calcification, healing bone tissue	[122]
	100 ng/animal	FVB and GPR18^−/−^ mice	Peritonitis	*E. coli*	↓ PMN recruitment ↑ efferocytosis ↑ PMN apoptosis	[123]
	200 ng/animal		Skin pouches	*S. aureus*	↓ PMN recruitment ↓ bacterial load	[123]
	100 ng/animal	FVB mice	Sepsis (CLP)	Polymicrobial	↓ IL-6, IL-1β, IL-23, TNF-α, IL-17, IL-10, PGE_2_ and LTB_4_ ↑ survival ↑ phagocytosis	[124]
RvD1 or AT-RvD1	50 ng/animal	FVB mice	Peritonitis	*E. coli*	↑ bacterial killing and accelerated onset of resolution ↓ antibiotic requirements ↓ IL-1β, IL-6, IFNγ	[10]
	150 ng/eye	Balb/c mice	Stromal keratitis	Herpes simplex virus-1 (HSV-1)	↓ corneal neovascularization ↓ severity of lesions ↓ neutrophils, Th1 and Th17 cells ↓ IL-1β, IL-6, IL-12, CXCL1, MCP-1, CXCL2, VEGF, MMP-9	[125]
	100 ng/animal	C57Bl/6J mice	Pneumonia	*E. coli or P. aeruginosa*	↑ phagocytosis/efferocytosis ↑ bacterial clearance	[126]
	100 ng/animal	Cftr^−/−^ mice	Cystic fibrosis (lung infection)	*P. aeruginosa*	↓ bacterial burden ↓ neutrophil infiltration improvement of clinical scores	[127]
	5 μg/kg	C57Bl/6J mice	Chagas disease	*T. cruzi*	↓ parasite load ↓ IFNγ, IL-1β ↑ IL-10 ↓ cardiac fibrosis	[128]
	100 ng/animal	C57Bl/6J mice	Sepsis (CLP)	Polymicrobial	↓ neutrophil infiltration ↓ apoptosis of CD3 + T lymphocytes ↑ survival ↑ bacterial clearance ↓ NF-κB phosphorylation	[129]
	20 ng or 100 ng/animal	C57Bl/6J mice	Upper respiratory tract infection	Nontypeable *H. influenzae*	↑ efferocytosis temporal regulation of inflammatory cytokines and enzymes ↓ weight loss, hypothermia, hypoxemia ↓ bacterial burden	[130]
RvD5	100 ng/animal	C57Bl/6J mice	Intestinal disease (to mimic enteropathogenic *E. coli* (EPEC) in humans)	*C. rodentium*	↓ bacteremia ↑ survival development of immunological memory	[131]
RvT1	50 ng (each)	Mice	Skin pouches	*S. aureus*	↓ bacterial titers ↓ leukocyte migration ↓ NET formation	[132]
RvT2						
RvT3						
RvT4						
PDX	300 ng/animal	C57Bl/6J mice	Sepsis (CLP)	Polymicrobial	↓ bacterial burden ↓ multiple organ injury ↑ survival ↑ phagocytosis ↓ TNF-α, IL-6 and MCP-1	[133]
	500–1000 ng/animal	C57Bl/6J mice	Sepsis-induced acute lung injury	Polymicrobial	↓ pulmonary edema ↓ leukocyte recruitment ↓ IL-1β, IL-6, TNF-α, and MCP-1 upregulation of PPARγ and suppression of NF-κB	[134]
PD1/NPD1	100 ng-1 μg/animal	C57Bl/6J, 12/15-LOX^−/−^ mice	Severe influenza	PR8 (mouse-adapted H1N1 influenza virus), 2009 H1N1 virus	↑ survival ↓ viral replication improvement of pulmonary functions	[135]
	300 ng/eye	C57Bl/6J mice	Stromal keratitis	Herpes simplex virus (HSV)	↓ neutrophil infiltration ↓ IL-6, CXCL1, CXCL-10, CCL-20, VEGF-A, MMP-2, and MMP-9	[136]
MaR1	1–100 ng/animal	Balb/c mice	Sepsis (CLP)	Polymicrobial	↑ survival ↓ TNF-α, IL-6, IL-1β ↓ AST, ALT, Cre, BUN, NF-κB activity ↓ mitochondrial damage	[137,138]
	10 ng/animal	Balb/cByJ and Lgr6^−/−^ mice	Respiratory tract infection	Respiratory syncytial virus	↑ amphiregulin ↓ viral transcripts ↓ IL-13	[139]

15-epi-LXA_4_: 15-epi Lipoxin A_4_; LXA_4_: Lipoxin A_4_; LXB_4_: Lipoxin B_4_; RvE1: Resolvin E1; RvD2: Resolvin D2; RvD1: Resolvin D1; AT-RvD1: aspirin-triggered Resolvin D1; RvD5: Resolvin D5; RvT1-T4: 13-series Resolvins; PDX: Protectin DX; PD1/NPD1: Protectin D1/neuroprotectin D1; MaR1: Maresin 1; AST: aspartate transaminase; ALT: alanine transaminase; Cre: creatinine; BUN: blood urea nitrogen; ↑: increased; ↓: decreased.

**Table 3:** Studies that verified endogenous production of SPMs upon administration of bacteria, viruses, or parasites.

	Infectious Agent	Model	SPMs Detected	Timepoint Analyzed	Peak	Method of Detection	Ref
Bacteria	*E. coli*	Peritonitis	RvD3	24 h after infection	N/A	LC–MS/MS and MRM	[172]
	*E. coli*	Peritonitis	MaR1	0, 4, 12, 24 h after infection	4 h	LC–MS/MS and MRM	[173]
	*E. coli*	Pneumonia	RvD1	0, 24, and 72 h after infection	72 h	LC–MS/MS and MRM	[126]
	*Clostridium butyricum* MIYAIRI 588	Antibiotic-Induced Dysbiosis	PD1	4 and 8 days after administration	N/A	UHPLC-Triple TOF/MS	[176]
	Polymicrobial	Sepsis	LXA_4_	6 and 12 h after CLP	6 h	LC–MS/MS	[118]
			LXB4		6 h		
			RvD1		6 h		
			RvD5		12 h		
Parasite	*Toxoplasma gondii*	Toxoplasmosis	LXA_4_	0, 5, 10, 15, 20, 25 days after infection	15th day	Commercial kit (Neogen)	[177]
	*Klebsiella pneumoniae*	Pneumosepsis	LXA_4_	6 and 24 h after infection	6 h	Commercial kit (Oxford Biomedical Research)	[116]
	*Toxocara canis*	Neurotoxocarosis	NPD1	7, 14, 28, 42, 70, and 98 days after infection	28th day (cerebrum), 98th day (cerebellum)	LC–MS/MS following negative electrospray ionization	[178]
	*Toxocara cati*				14th day (cerebrum and cerebellum)		
Virus	Influenza A virus strain A/Puerto Rico/8/34 (H1N1) (PR8 virus)	Severe influenza	PD1, LXA_4_	0, 6, 12, 24, and 48 h after infection	N/A	LC–MS/MS and MRM	[135]

## Data Availability

Not applicable.
